# Case of Unusual Determination of Blood to the Head

**Published:** 1820-11

**Authors:** H. B. Evanson


FOR THE LONDON MEDICAL AND PHYSICAL JOURNAL.

### *Case of unusual Determination of Blood to the Head.*

By H. B. Evanson, M.B. M.R.I.A.

MR. ——, æt. 23, of a nervous temperament, tall and slight, was seized, in November 1818, with a momentary stupor and loss of muscular power; his face being at the same time flushed and turgid. He had been subject, when a child, to occasional diarrhœa, with a discharge of blood, in small quantities, from the intestines. As he approached towards puberty, the affection of his bowels ceased, and he became liable to epistaxis on any irritation of his nose, from which blood flowed also sometimes spontaneously. This being so common an occurrence with boys, attracted no particular attention, until frequent returns of profuse hemorrhage became habitual. Early in the year 1817, an astringent wash was directed for him, and he was ordered to take two pills of aloes and myrrh every second morning generally, but to use them daily whenever he should feel the bleeding to be at hand; of which he was usually aware, by a slight aching and sense of fulness in his head. These measures he adopted for a short time, but speedily gave them up, on finding them unpleasant; and I heard nothing more on the subject until the occurrence of the attack in 1818.

In answer to my interrogations, he told me that the flow of blood from his nose had almost entirely ceased for some time previous to my seeing him; that he felt himself so ill a few minutes before, as to find it difficult to avoid falling. He complained also of his stomach being out of order, and said that his bowels were inclined to costiveness. Leeches, purgatives, (pil. coloc. comp.) a restricted diet, and regular exercise, were the measures then resorted to.

As he continued to complain of head-ache, he was recommended to have recourse to leeches a second time: he left Dublin, however, without complying with this advice.

The circumstances of this case made it necessary for me to write to the patient's family, in order to inform them of his illness, the nature of which I endeavoured to explain; telling them, that apoplexy or epilepsy would probably be the result of deviation from the plan of treatment laid down for him, and directing leeches to be provided, in order that they should be ready for immediate application, in case of a recurrence of the attack which I described, or of the continuance of head-ache.

Whilst in Dublin, the patient had three or four subapoplectic faintings, similar to that which I witnessed; and I found that he had been prevailed on to consult a physician of eminence, by whom the symptoms were attributed chiefly to derangement of the digestive organs. He remained in the country until the latter end of spring in the following year; but his life was miserable. Scarcely a week passed without one or more of these subapoplectic faintings, with a threatening of epistaxis occasionally, and a feeling as of strong pulsation and of weight in the head. Though the patient was following the prescriptions of another, I was compelled, under these urgent circumstances, to inform his friends that nothing but blood-letting would secure him from one or other of the results which had been predicted; but, unfortunately, they could not prevail on him to resort to it, and he was seized with a violent attack about the middle of the spring. He became quite insensible; his leg and arm of the left side were convulsed. From this state he was recovered, after some days, by purgatives, blistering, and the detraction of a small quantity of blood. The purgatives consisted, I believe, chiefly of calomel, as his mouth was slightly affected. On the expiration of a few weeks, he was again enabled to visit Dublin, where he put himself under the care of the physician whom he had before consulted. This gentleman now attributed the disorder to debility, and prohibited local or general loss of blood for the future; at the same time expressing regret that either had been at all adopted. The patient was directed to take carminative aperients every day: these, however, proved inadequate to secure natural discharges from the bowels. In addition, the cold shower-bath was ordered, by means of which the symptoms were for a time mitigated. Having returned to the country early in the month of July, he passed the remainder of the summer and the autumn by the sea-side, using the bath and the above-mentioned medicines every day. The fainting-fits were much diminished in frequency, but his head continued to annoy him occasionally: his sleep was often disturbed; and he now-and-then complained of coldness in his stomach after dinner. From the commencement of his illness, darkness had been unpleasant; and, latterly, until he had more society, was even intolerable to him; so much so, that he would even order four candles to be lighted together in his sitting-room. He complained, all the time that he was using these measures, of costiveness, attributing the deficiency of evacuations to the unyielding state of his bowels, rather than to the inadequacy of his medicines.

On the 10th of last November (1819), I received a summons to attend him. He had so severe an attack, that the physician who saw him before I was sent for had but little hope of his ultimate recovery. For about a week before its occurrence he felt it approaching, by the presence of pain and fulness in his head, accompanied with a threatening of epistaxis; also by languor and general irritability; but he had recourse to no other measures than those which he had been previously using.

His illness increased, and he was with difficulty able to reach his room from the bath, (a distance of about forty yards,) when he sunk on the floor. Convulsions supervened, confined, as I was informed, entirely to the left side. A surgeon-apothecary administered a purgative, shaved the patient's head, and opened the temporal artery; but, in consequence of the incision being made a little too high up, not more than a few ounces of blood flowed from the wound. These measures afforded some temporary relief, but not much. A blister was next applied to the neck, the purgatives were continued, and blood was again taken from the temple to the amount of about six ounces.

During the greater part of this time the patient lay in more or less of a comatose state, particularly towards evening, when there was an exacerbation of the symptoms, with convulsion or a tendency to it.

On the fourth day of the attack he was visited by a physician, who took away sang. ℥xiv. from his arm, with most decided benefit; applied a blister to the scrobiculus cordis; and prescribed pills of extrac. coloc. comp., pil. hyd., et pulv. ant. This gentleman quitted him the following morning, and I arrived towards the close of the day after.

I found the patient with a pulse at 80, full and soft; his face still retaining more than usual heat and redness. His head was yet somewhat full and painful; his left side was affected with an unpleasant numbness and tickling; and his tongue was foul, but not more so than was to be expected. His bowels having been affected but twice on that day, he was ordered ℥ iſs of the senna mixture, prepared with the usual proportion of neutral salt, of manna, &c. which shortly brought away several discharges. At night I gave him pulv. jacob. veri, gr. iv. with rhubarb. A similar quantity of both medicines was taken on the following day, with an additional dose of the powder at noon. He continued for some days under those measures, increasing the quantity of James's powder by a grain added every second day to each dose, until the amount became gr. viii.; at which time he was directed to take them only at night; his health being so much restored as to enable him to walk a mile without fatigue. The pain of his head, which, since the commencement of his illness, had been almost constant, now annoyed him only occasionally, and, when present, became much slighter; his rest, however, was not natural. He complained that his left side still retained a sensation of numbness; for which, as well as for a yellowish foulness on the posterior part of his tongue, I directed pil. hyd. ʒi. of which gr. x. were taken every second night, in addition to the remedies before prescribed. On leaving him at this time, I gave him directions to abstain entirely from animal food until I should see him again; to use the senna mixture, or other purgatives, (ordered for the sake of change,) every morning, so as to procure three or four evacuations daily; to persevere in the use of James's powder; to take exercise as often as the weather would permit; and to have two cupfuls of blood taken from his arm, in case he should feel another attack to be at hand: this experience had taught him, as well as his attendants, to be aware of, by the presence of symptoms which I have already enumerated.

Notwithstanding that he adhered strictly to my injunctions, it was found necessary to take blood from him twice within a fortnight after I quitted him. The first of these venesections took away, for two days, all unpleasant sensations, and afforded so much immediate relief, that he expressed himself strengthened by it, though the operation was performed on him whilst in a sitting posture. The repetition of it was oftener than I directed, and might possibly have advantageously given place to local depletion; but it eventually appeared to be of use, as it left him entirely free from tightness in the head, which had previously distressed him.

Two or three days after this, he left the remote part of the country where he had been, and came nearer to my residence. His head at the time was not much distressed, though occasionally uneasy. His tongue and pulse were natural, the latter indeed below 70; but he seldom rested comfortably until towards morning. He was, however, altogether pretty well; but weak from the effects of his illness, and of the depletion which he had undergone. I now permitted him to use broth of moderate strength, and substituted the preparation of the Pharmacopœia for James's powder. Of the latter he was not able to exceed gr. x. without exciting perspiration; the first dose of the former was therefore fixed at gr. vi. with directions as to its increase similar to those formerly given. His common purgatives, pil. coloc. comp. and the senna mixture, were changed for the following: ℞. Infusi sennæ; infusi gentianæ comp. (P. L.) utriusque, ℥iij.; tincturæ sennæ, ℥j.; sulphatis magnesiæ, ℥ij. ft. mistura. ℞. Aloes hepaticæ, ʒj.; assafœtidæ, ℈ij.; olii carui, gtt. xij.; sapon. venet. q. s. ut fiunt pilulæ viginti, sumat vel pilulas duas vel misturæ quod satis sit, mane quotidie.

These were discontinued after more than a fortnight's use, on account of their being disagreeable, and not appearing to possess any particular advantage for the patient over his former purgatives, which were again resorted to.

He continued till the latter end of January in the present year (for a space of two months), quite free from those faintings which used to harass him, regained his natural liveliness, and was able to take exercise on foot in the open air, even during the severe weather which prevailed at that time, adopting just sufficient precaution against wet and cold. The only thing particularly worthy of remark about him, was a considerable degree of that irritability which is in common language termed nervousness; any sudden noise would cause him to *bounce* from his seat. During this interval, fulness and pain of head became twice rather considerable, accompanied with a slight yellowish foulness of the tongue; but his pulse was not affected. These accessions subsided in the course of two or three days, on his taking pulv. ant. gr. iij. as an additional dose at mid-day, and using his purgatives so as to procure from six to eight evacuations daily. His pills were changed for the time to ext. coloc. comp. et pil. hyd. nearly equal parts.

At the latter end of January, when the frost suddenly ceased and gave place to moist and close weather, he was attacked with aching and tightness of his head, and with the depression of spirits which always attended those sensations. They were not at first very considerable; but, in consequence of their not yielding to active purging, together with the powders taken twice a-day, (of which he was not able to bear as much as he took during the cold weather, the extreme doses now being gr. iii. and ix. whereas before, on a similar occasion, they had been gr. iii. and xii.) I directed him, by letter, to have recourse to leeches; fifteen of which were accordingly applied, on the 22d, with immediate relief.

On the 24th his illness again increased; one of the *leech-bites* burst out bleeding, and he wet a small handkerchief with blood from his nose. He became very weak, and the gentleman who applied the leeches told me his pulse was nearly as high as 100. His feet were put into hot water at night; and he found himself better on the following day, (Jan. 25.)

On the evening of the 26th, I found him sitting by the fire; his pulse exceeding the natural standard only by a few strokes; his tongue showed but little foulness. He complained of tightness, heat, and pain of his head, with numbness of the left side. His countenance, indeed, would have told me that these sensations were present, for his face was flushed and perceptibly increased in fulness. As his bowels had been affected nine times on that day, I deferred, on account of the lateness of the hour, adopting any other measures until the morning, further than repeating the pediluvium.

On the 27th, his head was much distressed; he had passed a bad night; his pulse was full and accelerated. He was then ordered powders, consisting each ex. pulveris jalapæ, gr. viij.; submuriatis hydrargyri, gr. v.; pulveris aromatici, gr. ij.; one of which he took immediately. His hair was cut close; eighteen leeches were applied to his temples, and, after they dropped off, the flow of blood was encouraged for the space of nearly three hours. Just before the leeches were applied he had a tendency to fainting; but the detraction of blood brought relief from this and every other symptom. When the wounds were bound up, he said that he had quite recovered his strength, talked freely to those about him, and expressed himself to be better than he imagined he should be at the termination of three days. The purgative powder affected his bowels six times. He was directed to take one of these on each of the two succeeding mornings, and to go on as usual afterwards; except that he was to discontinue the antimonial powder, (the dose was just then gr. xv.) until I should order it to be resumed.

*Feb.* 12.—He had been quite well since I last saw him; but a recurrence of symptoms similar to those just described, milder however, made it now necessary to apply fifteen leeches. On the day following, a blister was placed to the occiput; the part was dressed with ung. sabinæ, and he was directed to keep up the discharge, when it should show a disposition to cease, by a temporary application of a blister. Both these days he took as a purgative one of the powders formerly directed, which affected him six or seven times each day.

On the 14th he felt himself well, except that his left leg retained an unpleasant feeling; his head was just not clear.

A good deal of general irritation being caused by the blistered part, it was healed shortly after, and the antimonial powder was resumed on the 18th, at gr. vij. as he thought himself better while using it. He was also directed to limit the action of his medicines to two evacuations in the twenty-four hours, while he should feel himself well; but on other occasions to use them as before.

*March 5th.*—A return of the usual symptoms made it necessary to have recourse to leeches again; not more than eleven fastened. The attack is partly attributed to fatigue, and to exceeding his usual diet.

*7th.*—On the morning of this day he had, while sitting down, an attack of insensibility, (the first for the last four months,) which lasted about ten minutes. During its continuance, his face did not lose its colour; and his pulse is described by his attendants as irregular, but for the chief part of the time very strong and quick. His dose of the antimonial powder was increased from gr. xiij. to gr. xv.; a terebinthinate emulsion was directed for him, to be taken every morning; and about five ounces of blood were taken from the back of his neck by cupping.

*April* 22.—Has been quite well since last report. He takes pulv. ant. ℈j. at night, which makes him hot without exciting perspiration. It sometimes acts on his bowels, so as to make it necessary to omit the turpentine mixture on the following day. Let the dose be diminished to gr. xv. He is anxious for permission to use leeches, as his head is not quite easy just at present. Applic. temp. hirud. xij.

*May* 26.—Continues well. Let the dose of the powder be gradually diminished.

*Sept. 8th.*—About the 1st of July, the patient commenced the use of the shower-bath, and omitted the powders; but the recurrence of uneasy sensations made it necessary to return to them after a few days. Since that time he has enjoyed good health, using both remedies in conjunction.

Remarks.—To some who may peruse the case which has been just related, it will probably appear strange that there should be any difference of opinion as to the plan of treatment which was pointed out by the symptoms. These, however, were sometimes ambiguous; and I have no doubt that professional skill and experience were misled for want of that information which the patient himself could not give.

From the earliest ages of medicine, we have been cautioned against the danger attending the suppression of wonted discharges. The cessation of such in the present instance caused the immediate symptoms, but the danger was antecedent to it. The history of the case shows that the patient was inclined to irregularities of the circulation from his childhood. That he was first liable to diarrhœa and discharges tinged with blood, was probably a matter of little moment; nor was the subsequent occurrence of epistaxis to be regarded as of much consequence; but a sedentary life, concurring with a certain disposition of body, tended to fix the habit of undue determination of blood to the head, which had well nigh ended in hemiplegia. As long as the vessels of the nose yielded to the increased impetus of the contained fluid, no great inconvenience resulted; but, when relief ceased to be obtained through the usual outlet, the brain became oppressed, and then ensued the alarming situation of the patient. Had he been accustomed to much bodily exercise, it is extremely probable that the congestive effort to the head would have been prevented by a more general distribution of the blood, depending on greater development of the muscular system.

Acidity and flatus in the stomach, which are regarded as marking the origin of the other symptoms, seem to have been but secondary. They were not at all overcome by means used in accordance with the former supposition; whereas they afterwards yielded readily to measures dictated by attention to the primary seat of disease. An inclination to attribute a variety of disorders to derangement of the digestive organs, is certainly too prevalent at the present time. Indeed, it seldom happens that improvements in medical science, arising from the labours of eminent individuals, are not alloyed with injury resulting from too great extension, and a consequent misapplication, of their observations, by themselves or by others. The most elevated of us are liable to errors of the kind; for the highest medical certainty is but probability. An over-extension, however, of the principle just noticed, is probably in most cases innocent, except in particular instances, such as the present, where it must lead to inert and ineffective practice. In the connected and complicated machinery of the human economy, disorder cannot exist to any extent without derangement in the functions of those organs which are directly subservient to nutrition. To them our remedies should be directed; and, whether they be the primary seat of disease, or that their deviation from the healthy state be consecutive, in an immense number of cases where disorganization has not taken place, the prospects of success will scarcely be affected by an error which can have but little influence on the treatment adopted by a physician who pays sufficient attention to his patient.

There are strong reasons to conclude, that the violent symptoms which have been detailed could have been prevented by adopting, at the commencement, that plan of cure which was afterwards productive of so much relief. At the very onset, measures would probably have sufficed, less active than those called for when lapse of time and the sufferance of two severe attacks, contributed to render the case more untractable. Even during the employment of the shower-bath, if topical bloodletting had been occasionally resorted to, and a moderate discharge kept up from the bowels, that powerful remedy might have had a very different effect. Immediate relief has been only obtained by detraction of blood; but, as this tended to perpetuate the habit which required to be subdued, it became necessary to resort to other means, in order to obviate the necessity of having recourse to leeches or to the lancet.

In altering habits that have been already formed, the remedies are principally directed to the capillaries or extreme vessels, the laboratories of the system, as it is on them the salutary change is to be induced. The high character of antimonial powder pointed it out as a powerful auxiliary in this case; and the detail shows that its employment was attended with well-marked benefit, though the violence of the symptoms, and their mixed nature, seem to have prevented it from being so effectual as it is reported to have been in other instances. The circumstances of the foregoing case did not allow me to observe any difference in action between the antimonial powder of the College and the patent medicine, except in point of strength. The greater activity of the former appears to me to give it a sufficient claim to the preference in cases of any violence; for medicines of different power, though mutually proportioned so as to establish a parity of strength in the dose, are frequently followed by a marked inequality of salutary effect.

The powder was discontinued at one time, as I wished to see how the patient would go on without it; but he returned to it, without my knowledge, in a few days after the order for discontinuation. The true interval of its omission is to be counted from the 12th to the 18th of February. The length of time during which the patient has been taking this medicine, (more than nine months,) and the extent of the doses, furnish an additional proof of its safety.

In such a case as the foregoing, reliance cannot be placed on any one subsidiary remedy. Purgatives and other means were therefore used with considerable activity. Mr. Hunter has asserted, that purging lowers action without diminishing strength. The assertion is a general one, and as such I never have had occasion to doubt its truth, either while witnessing the use of purgative medicines in the hands of active practitioners, or subsequently judging from my own immediate experience. The improved mode of treatment in typhus, is a sufficient proof of its correctness; and yet, I think, writers on Fever (with the exception of a few, amongst whom Dr. Armstrong's name suggests itself to me,) have not considered the matter in the best point of view. The mere removal of fecal matter is but a part of their office. They seem to allay disturbances of the system, not so much by expelling one source of irritation, as by establishing a stimulus of a different kind, the degree of which should be regulated according to the nature and extent of existing disorder. To keep up a discharge of the mucous, the biliary, and the pancreatic secretions, is to the system at large more than a topical application is to an affection purely local.

How far the nervous system is concerned in undue distribution of blood to the cerebral organs, it is impossible to decide: nor can we say in such cases, (if we should admit the relation of cause and effect to exist between them,) what state of the former it is that produces vascular derangement. To assume that it is a state of debility, is contrary to physiological analogies, and is fraught with practical danger.

In the present instance, local plethora seems closely connected with nervous disorder. As a matter of opinion, I should myself say, that the connection was chiefly reflective.

Whether to break the chain of influence, or to overcome a cause of the disease, it was evidently necessary to take the nervous system into account in prescribing. It was with this view the oil of turpentine was ordered, which seems to have caused, in part, the improved state of health which the patient has enjoyed during the last five months. With the powers of this medicine, as well as of others, we have latterly become better acquainted ; used as a purgative, it may prove a useful auxiliary in the treatment of many cases to which the antimonial or James's powder is applicable.

I do not recollect to have seen a case detailed which I would say is similar to the foregoing. The nearest approach to it occurs in Morgagni;* but the two cases related by him, to which I refer, differ from it in many circumstances, though they agree with it, particularly the last, in the leading symptom.

*8, South-Mall, Cork; Sept.* 14, 1820.

* *De Sed. et Caus.* Epist. iii. art. 24; Epist. ix. art. 25.

